# A new day dawning: *Hemerocallis* (daylily) as a future model organism

**DOI:** 10.1093/aobpla/pls055

**Published:** 2012-12-21

**Authors:** M. J. Rodriguez-Enriquez, R. T. Grant-Downton

**Affiliations:** 1 Instituto de Bioorgánica Antonio González (IUBO), University of La Laguna; Avenida Astrofísico Francisco Sánchez, 38206 La Laguna Tenerife, Spain; 2 Department of Plant Sciences, University of Oxford, South Parks Road, Oxford OX1 3RB, UK

**Keywords:** Asparagales, daylily, flower opening, medicinal plant, model organism, programmed cell death, self-incompatibility.

## Abstract

Genetic model organisms have revolutionized science, and today, with the rapid advances in technology, there is significant potential to launch many more plant species towards model status. However, these new model organisms will have to be carefully selected. Here, we argue that *Hemerocallis* (daylily) satisfies multiple criteria for selection and deserves serious consideration as a subject of intensive biological investigation. Several attributes of the genus are of great biological interest. These include the strict control of flower opening and, within a short period, the precisely regulated floral death by a programmed cell death system. The self-incompatibility system in *Hemerocallis* is also noteworthy and deserves more attention. Importantly, the genus is widely cultivated for food, medicinal value and ornamental interest. *Hemerocallis* has considerable potential as a ‘nutraceutical’ food plant and the source of new compounds with biomedical activity. The genus has also been embraced by ornamental plant breeders and the extraordinary morphological diversity of hybrid cultivars, produced within a relatively short time by amateur enthusiasts, is an exceptional resource for botanical and genetic studies. We explore these points in detail, explaining the reasons why this genus has considerable value—both academic and socio-economic—and deserves new resources devoted to its exploration as a model. Its impact as a future model will be enhanced by its amenability to cultivation in laboratory and field conditions. In addition, established methods for various tissue and cell culture systems as well as transformation will permit maximum exploitation of this genus by science.

## Abstract (precis)

In this point of view paper, we argue that the monocot genus *Hemerocallis* (daylily) satisfies multiple criteria for selection as a ‘new model organism’ for intensive biological investigation. We discuss its important and interesting attributes at the biological, horticultural and medicinal levels. These include an intriguing self-incompatibility system, a sophisticated mechanism for flower bud opening and programmed floral death, and a long history of use by man as a vegetable, ornamental and medicinal plant. We examine the potential for modern technical developments to transform *Hemerocallis* into a valuable model plant.

## Introduction

Studies of model organisms have revolutionized biological and medical sciences. Plant biology has made profound progress since the primary dicotyledonous plant genetic model—thale cress, *Arabidopsis thaliana*—was adopted as a convenient laboratory study organism. This species exemplifies the merits of the primary model organism approach. The comparative speed and simplicity of its life cycle and development, as well as its compact size and relative ease of handling in experimental work, made it the preferred choice as a study organism in hundreds of plant sciences laboratories. As a self-fertilizing, in-breeding species with a relatively small genome, its genetics were approachable and consequently it was the first plant genome to be sequenced. Although not economically important in itself, this species was morphologically and physiologically representative of many other dicots, especially numerous important crop plants in the Brassicaceae. These attributes have allowed *Arabidopsis* to generate, with astonishing rapidity, exceptional new data for plant scientists that is broadly applicable to many other species.

Owing to their exceptional economic benefits, other plants with much larger genomes, such as maize (*Zea mays*) and rice (*Oryza sativa*), have been extensively studied in a similar way, as techniques that permit their investigation at the molecular level have improved. Today, such technical advances are generating exciting potential to adopt many other species as new ‘model’ plants. The most significant advance has been the development of high-throughput sequencing of genomic DNA and RNA populations, such as Illumina sequencing methods (Deschamps and Campbell 2010). Other improvements to sequencing methodology, such as direct sequencing (Deschamps and Campbell 2010), are likely to further change plant biology. The improvements in computational methods that permit assembly of the short sequences into an entire genome sequence have also been dramatic. Together, these advances have allowed the huge genome sequence of allohexaploid bread wheat (*Triticum aestivum*) to be published (Brenchley *et al.* 2012). Other technical advances such as improved methods for genetic transformation, and many new transformation-dependent reverse genetics techniques—such as efficient, specific gene silencing via artificial microRNAs (Ossowski *et al.* 2008) and the use of zinc-finger nucleases to engineer specific nucleotide changes in plant DNA sequences (Weinthal *et al.* 2010)—are opening up new frontiers in the rapid analysis of gene functions.

Nevertheless, the choices for ‘new model organisms’ in this new ‘post-*Arabidopsis*’ genomic era have to be refined and well informed, to ensure that finite resources are allocated appropriately. Ideally, several different criteria need to be satisfied if a plant taxon is to be selected for such intensive studies. Firstly, the proposed model taxon would have a set of characteristics or attributes that when studied would answer or illuminate important biological questions. In this respect, a balance is required between the model organism exemplifying its phylogenetic group and having unusual or even unique phenotypic traits of exceptional biological interest. Secondly, the relevance of the proposed taxon to contemporary agricultural, horticultural and medicinal usage—and hence an economic justification for its selection—must be taken into account. Finally, for practical purposes, the ideal study species would be a taxon that is easily cultured and manipulated under laboratory conditions, for example amenable to tissue culture and transformation procedures. When all of these are considered, it should become a great deal easier to select appropriate new model organisms for plant science.

In this point of view article, we discuss why one monocotyledonous taxon, *Hemerocallis* (daylily) of the Asparagales, satisfies all of these criteria admirably and in our opinion deserves to be seriously considered as a model organism of the future.

## Botanical background

**Fig. 1:**
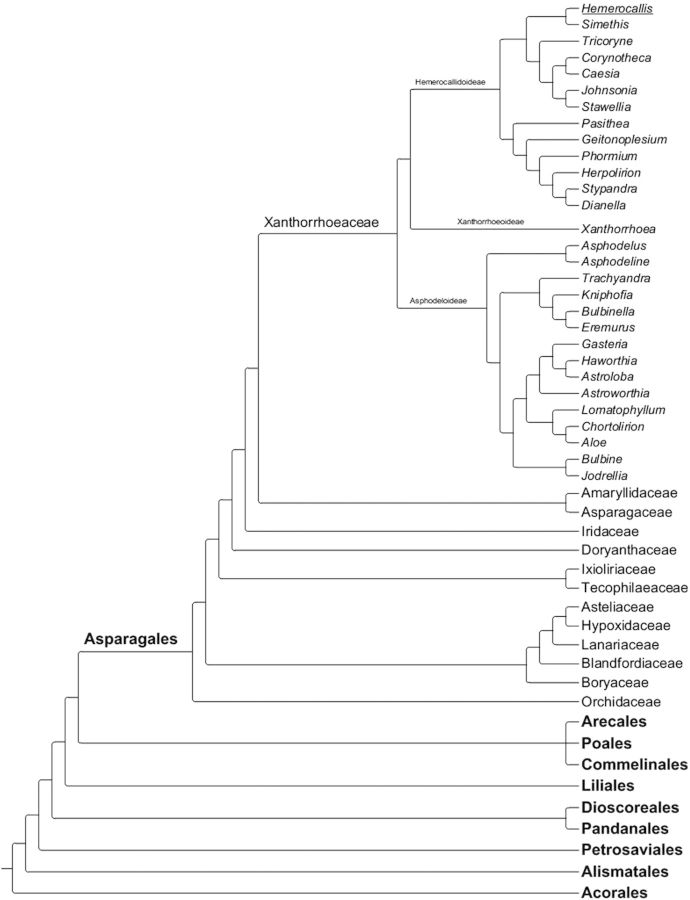
**Phylogeny of monocots following APG III classification, showing a zoom on the order Asparagales to the level of family and a zoom on the Xanthorrhoeaceae (*sensu lato*) to the generic level.** The phylogeny was created using the Phylocom program based on published phylogenies (Webb *et al.* 2008). See the text for a further discussion of the *Hemerocallis* classification.

The genus *Hemerocallis* was first classified as a member of the lily family in its broadest sense—the Liliaceae *sensu lato*. However, 20th century treatments of the monocotyledonous plants recognized that this classification was actually flawed and *Hemerocallis* was placed in a separate family, Hemerocallidaceae (Dahlgren *et al.* 1985). Recently, molecular studies have shown evidence supporting this placement of the genus away from the Liliaceae *sensu stricto*. Despite the great resolution that such studies provide, the exact position has remained rather problematic to determine. Initially, molecular work placed *Hemerocallis* in the Hemerocallidaceae (Angiosperm Phylogeny Group (APG) 1998, 2003), but subsequently the genus was sunk into the Xanthorrhoeaceae (*sensu lato*) (grass tree family) by APG III (APG 2009), which combined the Hemerocallidaceae, Asphodelaceae and Xanthorrhoeaceae *sensu stricto* (Fig. 1). The most recent molecular treatment resurrects the family Hemerocallidaceae (Seberg *et al.* 2012). While the genus itself is easily delimited (Dahlgren *et al.* 1985), the taxonomy of the genus remains rather confused and there has been no recent formal monographic treatment of the genus. The primary monographic study of the genus was published by Stout (1934), although several new species have been described since then, e.g. *Hemerocallis taeanensis* (Kang and Chung 1997). It is now obvious that the genus will pose many taxonomic problems. One Japanese endemic taxon, *Hemerocallis citrina* var. *vespertina*, is likely to represent convergent evolution in three independent lineages based on molecular and morphological data (Noguchi and Hong 2004). There is clear evidence that the recognized species can hybridize in the wild (e.g. Kawano 1961; Hasegawa *et al.* 2006) and in cultivation there appear to be minimal barriers to hybridization between the different species (Stout and Chandler 1933; Stout 1934). However, a level of reproductive isolation in hybrids between *Hemerocallis fulva* and *H. citrina* was found in F_1_ backcrosses, most likely due to post-zygotic lethality (Yasumoto and Yahara 2008). The taxonomy of the genus is likely to be further complicated by extensive cultivation by humans since ancient times (Stout 1934; Xiaobai 1996; Valder 1999).

**Fig. 2:**
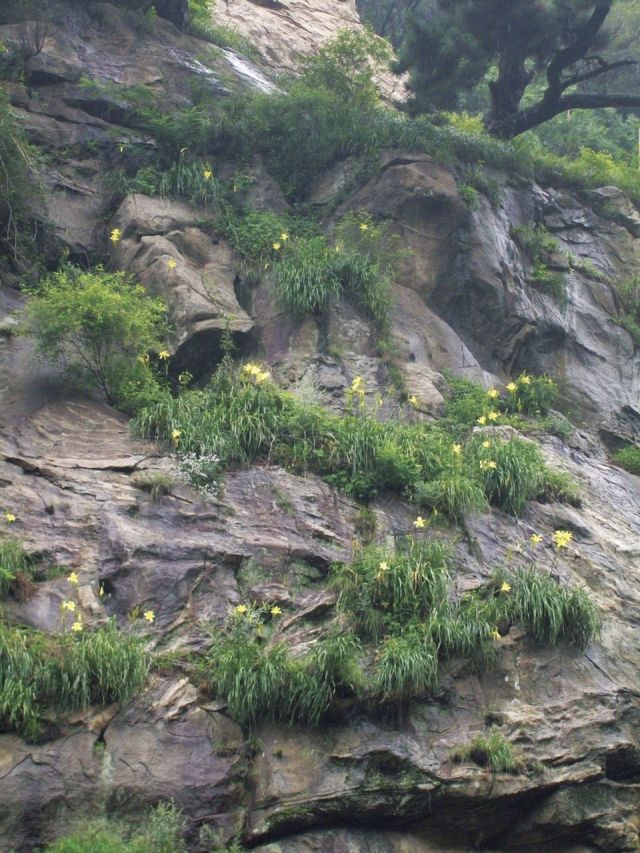
**Wild populations of *H. citrina* growing on cliff faces of Mount Tai, Shandong Province, China**.

The natural range of *Hemerocallis* encompasses temperate and sub-tropical Asia, with the main diversity of the genus centred on China, Korea and Japan. However, members of the genus are prone to become persistent, even aggressive, aliens in a relatively short space of time after introduction to new localities, for instance *H. fulva* in North America (Ehrenfeld 2008). Whether the natural distribution of *Hemerocallis* extends to Europe remains puzzling (Webb 1980); it is likely that *Hemerocallis lilioasphodelus* is a persistent, casual alien in most if not all European locations, rather than being truly native (Pyek 2003). These rather disjunct European populations of *H. lilioasphodelus* deserve investigation to determine whether they are genetically distinct from Asian *H. lilioasphodelus.* In general, the members of the genus are normally found in mountainous and grassland habitats (Fig. 2), but some species grow in other habitats such as on sea cliffs, e.g. *Hemerocallis hongdoensis* (Chung and Kang 1994).

**Fig. 3:**
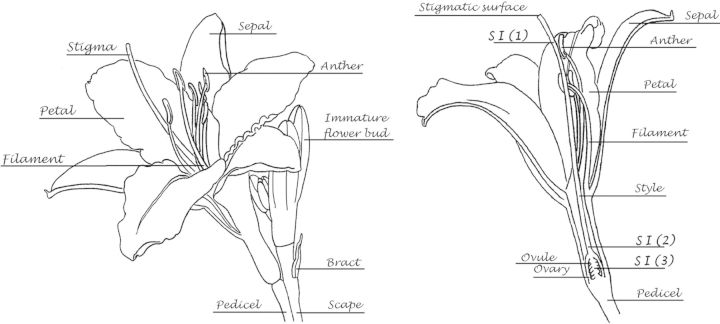
**Basic floral anatomy of *Hemerocallis***. The uppermost part of a flower scape with mature, open flower and developing buds is illustrated on the left. On the right, the diagram shows a longitudinal cross-section through an open flower. The locations of the different components of the SI system are shown: (1) the uppermost region of the stigma, at the entrance to the stylar canal; (2) the junction between the ovary and style; (3) within the ovary, post-fertilization. See the text for a further discussion of the SI systems in *Hemerocallis*.

Morphologically, the members of the genus *Hemerocallis* are relatively uniform (Stout 1934; Dahlgren *et al.* 1985). All share a herbaceous vegetative habit, with distichously arranged long, linear leaves arising from a perennial, fleshy rhizome at or just below ground level. The rhizome produces fleshy roots, often forming highly thickened tuberous reserve structures. The showy flowers are borne on short pedicels on few- to many-flowered erect inflorescences (scapes) (Fig. 3). In the species, the lily-like flowers consist of a trumpet-shaped corolla with six tepals (three sepals and three petals). The six stamens are conjoined to tepal tissue near the base of the corolla. The gynoecium is tripartite, with a long, thin, partially hollow style projecting the stigmatic surface beyond the stamens. As may be expected from their structure, the flowers are pollinated by insects such as moths and butterflies (Hasegawa *et al.* 2006; Yasumoto and Yahara 2006; Nitta *et al.* 2010; Hirota *et al.* 2012). The leathery ovoid to triangular seed capsules contain a small number of hard-coated seeds that are released as the capsule dries and splits.

## Special attributes of biological interest

There are two significant and special features of *Hemerocallis* that particularly recommend this genus should deserve serious consideration as a future model organism. Both are reproductive features of the genus that have already received the attention of scientific researchers.

The first is the strict developmental control of flower opening and flower senescence in *Hemerocallis.* All known species in the genus share the characteristic of rapid opening of the mature flower bud which occurs over a period of a few hours. This is followed—within 24 h of opening—by highly regulated, programmed death of key floral tissues (the sepals, petals and anther filaments). Indeed, both the common name and the generic name (literally translated as ‘day beauty’) come from this regular and predictable behaviour of floral death. Although not unique among flowering plants, the genus has an excellent track record in studies of the mechanisms that control opening and subsequently death of the floral tissues. The primary mechanism for opening of a mature flower bud would appear to be a hydraulic mechanism based on the action of hydrolytic enzymes on stored carbohydrates (Bieleski and Reid 1992). As might be expected, late bud development is accompanied by linear accumulation of dry weight but this is not matched by wet (fresh) weight, which increases disproportionately just prior to flower opening. At this time, the trisaccharide fructan that has accumulated would appear to be rapidly hydrolysed to fructose and glucose, driving water uptake and petal expansion. This physiological mechanism, dependent on fructan synthesis and then its hydrolysis, must be developmentally regulated by systems tightly linked to circadian rhythm regulation. Indeed, the timing of flower opening varies between species. This has been studied in natural hybrids between the nocturnally opening, moth-pollinated *H. citrina* and the diurnally opening, butterfly-pollinated *H. fulva* (Nitta *et al.* 2010). The timing of floral opening was determined to be a trait that was predominantly under the control of a single major locus, although other loci were likely to make quantitative contributions to this trait. However, the exact molecular nature of the regulation of the timing of bud opening remains mysterious. Nitta *et al.* hypothesize that the locus in the nocturnal *H. citrina* has a promoter to which an evening-phase oscillator (similar to TOC1) binds, while in morning-opening *H. fulva* the promoter has a morning-phase oscillator-binding motif. This study also provided clear evidence that another major locus controlled the timing of flower closing. Hence, just two loci may regulate the strict temporal behaviour of floral biology of *Hemerocallis*.

**Fig. 4:**
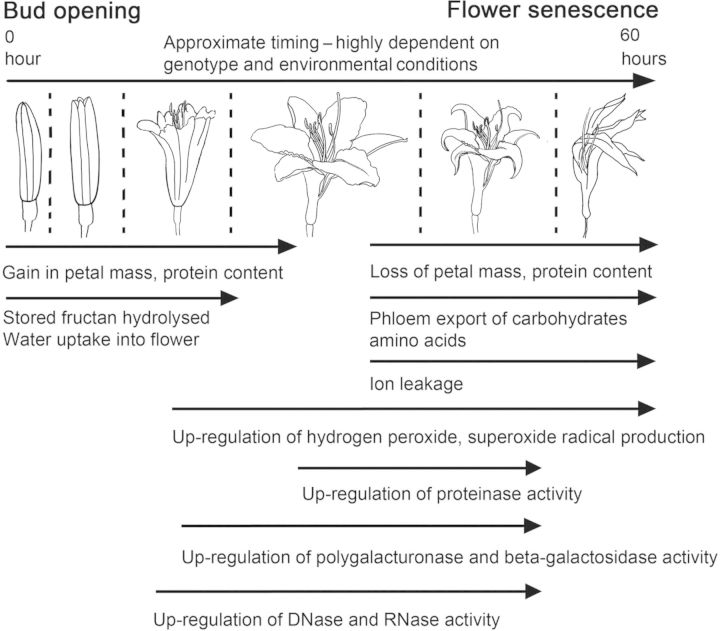
**Schematic diagram showing the approximate timing of key physiological and biochemical events that regulate the opening of a mature bud to senescence of the flower, taking place over c. 60 h**.

The mechanism of programmed cell death (PCD) has been much more extensively explored at the physiological and molecular levels. The system is intrinsic, as detached flowers and pieces of floral tissue are able to autonomously undergo PCD with the same timing. It is also an active system of PCD, dependent on protein synthesis, as an inhibitor of translation, cycloheximide, is able to inhibit both flower opening (Bieleski and Reid 1992) and PCD in *Hemerocallis* (e.g. Stephenson and Rubinstein 1998; Mahagamasekera and Leung 2001). There is ample evidence that this PCD mechanism involves a number of complex biochemical pathways that bring about systematic cell death in floral tissues. Several studies have implicated proteolytic pathways (Valpuesta *et al.* 1995; Guerrero *et al.* 1998; Stephenson and Rubinstein 1998; Mahagamasekera and Leung 2001) with active increases in various proteolytic enzymes after flower opening. Enzymes that modify cell walls, such as polygalacturonases and β-galactosidases, are up-regulated after flower opening and may contribute to the PCD (Panavas *et al.* 1998). The production of reactive oxygen species and disintegration of membrane integrity through lipoxygenase activity are also likely to make a major contribution to PCD (Panavas and Rubinstein 1998). Again, such oxidative changes are brought about by different routes—the levels of protective antioxidants such as ascorbic acid decrease as the flower ages, while peroxidase and superoxide dismutase activities increase. Finally, this senescence system can be truly classified as PCD as it is accompanied by significant DNA laddering by nucleases and also by up-regulation of RNases (Panavas *et al.* 2000). The main biochemical and physiological events of bud opening and flower senescence are summarized in Fig. 4.

Detailed knowledge of the PCD systems in plants remains biologically important. For example, PCD systems activated in tissue and organ development have significant effects on their eventual outcome, such as in anther development where tapetal cells surrounding developing pollen must undergo PCD (Parish and Li 2010). Furthermore, PCD is an essential ‘damage limitation’ and defence response in plants to invasion by pathogens and parasites (Greenberg 1997). It is interesting to note that the floral PCD mechanism in *Hemerocallis* cannot be easily broken; despite extensive horticultural breeding, there would appear to be no example to date of variants that have lost this mechanism. This unexpected strength of the PCD system in *Hemerocallis* has led Nitta *et al.* (2010) to propose that the major locus regulating floral closing and PCD at some fundamental level(s) must be necessary for plant viability, and it is likely to encode a multifunctional transcription factor.

This highly regulated system of flower opening and closing now deserves the full treatment of modern systems biology, to build a comprehensive picture of how this process occurs, integrating transcriptomic, proteomic and metabolomic data. Although the final result is a rapid destruction of an organ, the process is also co-ordinated with the rest of the plant as significant retrieval of resources takes place via phloem export (Bieleski 1995). How the signalling systems operate *in planta* to control these fluxes in flower opening and closing over just a short period of time itself deserves to be studied.

The other reproductive feature of *Hemerocallis* that has received far less attention, yet nevertheless remains another fascinating area for exploration, is the self-incompatibility (SI) system. Early work on the breeding systems revealed that a number of the species have a level of SI ranging from strong to weak (Stout and Chandler 1933). This early work is difficult to interpret but its strongest message is that SI in this genus involves complex, multi-level systems to prevent self-fertilization. There is evidence for an early system of SI where incompatible pollen tubes grow slowly and do not advance into the stylar canal. Intriguingly, and rather exceptionally, the main element of the SI system in *Hemerocallis* acts very late. This mechanism permits the germination and growth of incompatible pollen tubes through the style, until around the junction between style and ovary. Remarkably, the typically fast-growing pollen tubes of both compatible and incompatible genotypes are then held in near-stasis for a period of hours at this position, with only the compatible pollen tubes growing into the ovary to complete fertilization of the ovules. Unfortunately, anything other than basic histological details of this mechanism remain mysterious. For example, it is not known whether—in common with the gametophytic SI systems of *Papaver* and *Nicotiana* (Franklin-Tong and Franklin 2003)—the incompatible pollen tube is rendered inviable. If the incompatible pollen tubes are not incapacitated in this way by the SI system, it is possible that they are unable to respond to specific physical and/or chemical cues produced by the female tissues at this stage. What is so unusual is that both compatible and incompatible pollen tubes are, it would appear, often delayed in their progress towards the target ovules. In many other flowering plant species, pollen tube growth is relatively continuous and there is no lengthy ‘pit-stop’ prior to fertilization. Indeed, the great selective power of the competitive ‘race’ between an excess of pollen tubes to reach a limited number of ovules through the stigmatic tissues is a central tenet of the ‘progamic theory’ to explain the evolution of the flowering plants themselves (Mulcahy 1979). It may be that, post-pollination, changes in cell wall-modifying enzymes in the gynoecium may be responsible (Konar and Stanley 1969). Stout and Chandler (1933) did record cases where pollen tubes grew without delay to the ovary and ovules, and yet another level of SI (perhaps preventing fertilization or involving post-zygotic abortion) prevented offspring from being formed by self-pollination. The locations of SI phenomena are shown in Fig. 3.

The largely forgotten research on this particularly complex SI system deserves to be given further experimental attention today using the full raft of modern techniques. What signal(s) force the compatible pollen tube to cease elongation and how the growing tip of the tube is maintained in a plastic state that can be activated into further extension growth (Taylor and Hepler 1997) remain interesting questions. Furthermore, whether there is any connection between the floral PCD system and the operation of the SI system in the gynoecium would be interesting to determine. It is intriguing to note that most horticultural hybrid daylilies have retained a strong SI system that would appear to reject self-pollen effectively (R. T. Grant-Downton, pers. obs.). Perhaps this retention is due to the complexity of SI within the genus, with different stages at which SI phenomena can manifest.

## Economic justifications for selecting it as a model

**Fig. 5:**
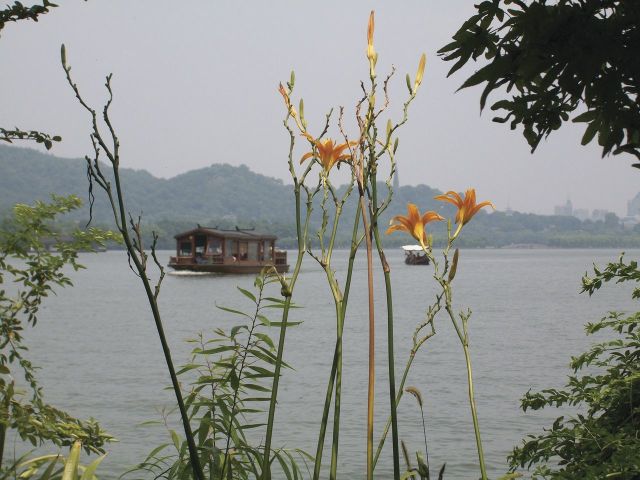
**Cultivated *H. fulva* growing in the gardens of West Lake, Hangzhou, China**.

*Hemerocallis* has a remarkable track record of domestic usage by humans. There is evidence that points to the genus being cultivated in China for several thousands of years (Valder 1999) (Fig. 5). The flower buds appear to be the most widely consumed (Mlček and Rop 2011), with young shoots and roots also being edible but the roots showing some toxicity (Zhang *et al.* 2004). Severe toxicity of *Hemerocallis* has occasionally been reported after ingestion of root material (Xiao *et al.* 2010). This is probably due to the presence of stypandrol (hemerocallin) in root tissues, a neurotoxic compound also found in the related genera *Stypandra* and *Dianella* (Wang *et al.* 1989). The flower buds are quite commonly used as a vegetable in the Far East; for example, they are dried and then incorporated into soups and stews, but can also be eaten uncooked. Several studies have investigated the properties of flower buds, for instance revealing that they are rather enriched with antioxidants compared with many other vegetables (Bor *et al.* 2006). The flowers contain a novel naphthalene glycoside, stelladerol, which has potent antioxidant qualities, as well as other antioxidant glycosides (Cichewicz and Nair 2002). Several caffeoylquinic acid derivatives in the flowers have also been identified as significant antioxidants (Lin *et al.* 2011). *Hemerocallis* flowers have considerable potential as ‘nutraceutical’ foods (Mlček and Rop 2011; Rop *et al.* 2012).

The genus has been considered to have exceptional medicinal value in its natural range (Mlček and Rop 2011). Traditionally, *Hemerocallis* has been used to treat sleep disorders and to alter mood; in Japan and China it is known as ‘forget one's sorrow plant’. These purported qualities have been scientifically tested and there is positive evidence for crude plant extracts to alter sleeping patterns (Uezu 1998) and alleviate depression (Gu *et al.* 2012; Yi *et al.* 2012) in animal models. The leaves have also been used to treat jaundice and inflammation, and Zhang *et al.* (2004) have shown that the leaves contain several compounds, such as roseoside, which strongly inhibit lipid peroxidation. The traditional use of *Hemerocallis* against schistosome infection has been supported by Cichewicz *et al.* (2002), where several compounds from roots, including novel kwanzoquinones, were found to be active against *Schistosoma* parasites. Anti-proliferative effects and cytotoxicity to cancer cells were also reported for several known and novel anthraquinones isolated from roots (Cichewicz *et al.* 2004).

Nevertheless, connecting specific compounds in *Hemerocallis* to therapeutic effects in humans largely remains to be achieved. Adopting *Hemerocallis* as a model organism would greatly assist the studies of biomedically active compounds. By generating more detailed molecular datasets for *Hemerocallis*, in tandem with biomedical studies of greater resolution to identify the active compounds responsible for therapeutic effects, *Hemerocallis* could be transformed into a substantial resource for the discovery of novel pharmaceuticals. Intensive studies would be economically and socially beneficial if obtaining new useful drugs is the goal.

## Exploitation of a horticultural heritage

While there is already an overwhelming argument for adopting *Hemerocallis* as a model plant, the horticultural heritage of just over a century of intense interest by Western gardeners adds further credence. At the most simplistic level, the popularity of *Hemerocallis* in gardens and its consequent economic value in the horticultural trade make its selection as a future model more appropriate. Additional to this economic justification, new and interesting possibilities for biological investigations were opened up by the activities of gardeners in hybridizing and selecting *Hemerocallis.*

Outside of the Far East, *Hemerocallis* is primarily cultivated as a garden ornamental, with currently very little interest in culinary and medicinal usage. This is despite horticultural material—including presumed Far East cultigens, the virtually sterile triploid forms of *H. fulva*—having been cultivated for centuries (Valder 1999). Although one of the earliest plants of the Far East to reach the gardens of Western Europe, serious horticultural attention only began after George Yeld made the first artificial interspecific hybrid, which precipitated a new era of deliberate breeding (Stout 1934). Subsequently, selection of the fertile hybrid breeding lines resulting from interspecific crosses, and the discovery and introduction of new species and variants to add to the gene pool, generated a complex hybrid gene pool. Stout of New York Botanic Garden was not only responsible for describing new species and maintaining them in cultivation, but also for a large amount of experimental hybridization work (Stout and Chandler 1933; Stout 1934). By repeating artificial crosses many hundreds of times, Stout was able to incorporate the genetics of almost sterile triploid clones of *H. fulva* (Chandler 1940) into hybrid lines. The origin of these old triploid clones is fascinating and mysterious in itself. No tetraploid individuals have ever been found in nature (Xiaobai 1996) and anyway there are significant barriers to interploidy crosses in *Hemerocallis*, with triploids only reliably produced from diploid–tetraploid combinations via *in vitro* embryo rescue (Arisumi 1973; Li *et al.* 2009). Presumably, they were formed by extremely rare events involving unreduced gametes; based on cytology, one investigator (Xiaobai 1996) has suggested that one double-flowered *H. fulva* triploid clone is an allotriploid hybrid and not an autotriploid.

**Fig. 6:**
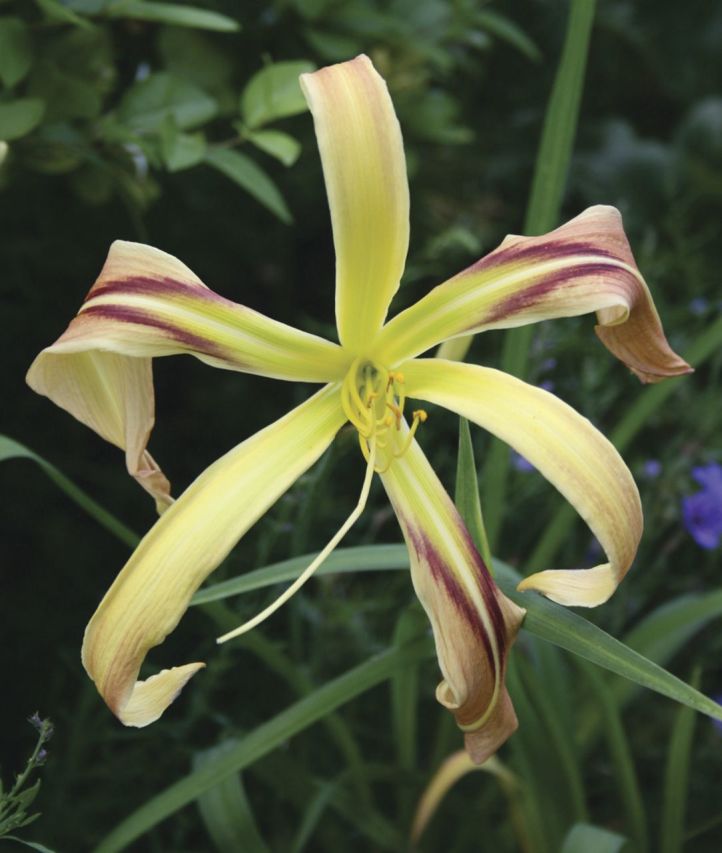
**A modern *Hemerocallis* cultivar, ‘Aldersgate’, of the ‘spider’ form showing the very long, slender tepals typical of these types**.

**Fig. 7:**
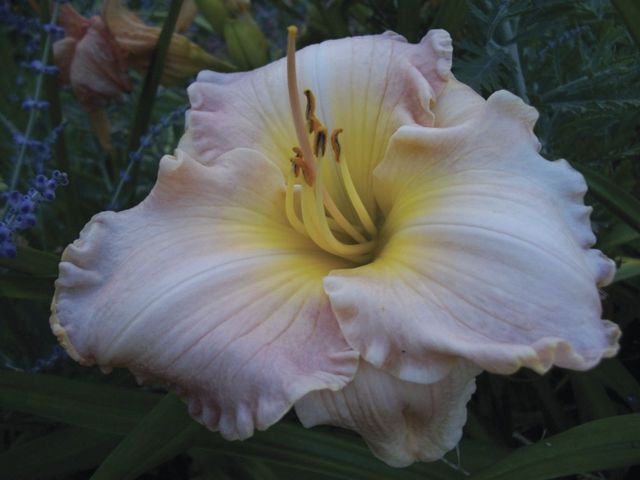
**A modern *Hemerocallis* cultivar, ‘Laura Nyro’, showing the effect of selection for shallow, open flowers with more rounded, blunter tepals**.

By the 1950s, this pioneering work meant that the gene pools of hybrid lines in cultivation contained a number of species such as *Hemerocallis altissima*, *H. citrina*, *H. fulva* and *H. multiflora*. Intensive selection of the fertile hybrid lines—primarily by horticulturists in the USA—resulted in great changes to plant characteristics, in particular to the floral morphology, with astonishing rapidity. Transgressive characteristics resulted, such as alterations to the placement and dimensions of the tepals—plant breeders selected away from the original lily-like, funnel-shaped to trumpet form of the species to generate variants with long, linear floral segments (the so-called ‘spider’ forms; Fig. 6) and, at the other extreme, more flat-opening flowers with rounded, broad and blunt tepals (Fig. 7) (Petit and Peat 2000, 2004). From the 1950s onwards, diploid hybrid breeding lines were joined by man-made tetraploids generated by treating plant material with anti-mitotic agents such as colchicine (e.g. Traub 1949; Arisumi 1964). These tetraploid lines then became increasingly popular in horticulture (Petit and Peat 2000, 2004). Since the initial period of interspecific hybridization in cultivation during the late 19th to early 20th century, led by Yeld and Stout, the majority of horticulturists working with the genus have simply continued to extend existing fertile hybrid lines rather than back-crossing to the original parental species or making new hybrids with more recently described species. Nevertheless, the original progenitor clones of the species used to generate hybrid lines have been maintained in cultivation due to their long-lived perennial nature. Tomkins *et al.* (2001) exploited this valuable horticultural resource, comparing the genetic diversity of the progenitor species and their primary hybrids with hybrid cultivars generated further down the line of intensive breeding using the amplified fragment length polymorphism technique. The species and early hybrids showed the highest genetic diversity, with genetic diversity progressively dropping from the cultivars in the mid-20th century to those produced in the late 20th century. As might be predicted, the tetraploid breeding lines displayed less genetic diversity than diploids. This work clearly shows that the intense artificial selection of plant hybrids recently taken up for ornamental domestication soon generates a measurable decline in genetic diversity. The genus offers a marvellous opportunity to investigate the effect of man-made hybridization and extreme artificial selection pressures during domestication on the genome and gene expression.

**Fig. 8:**
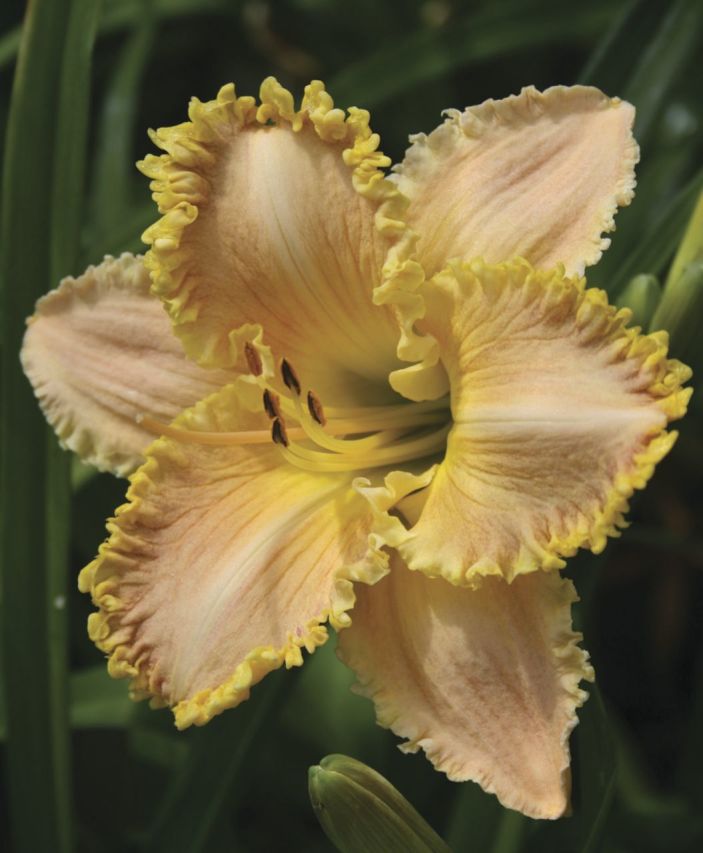
**A modern *Hemerocallis* cultivar, ‘This Side of Paradise’, showing the effect of selection for outgrowths of tissue at the edges of the tepals**.

**Fig. 9:**
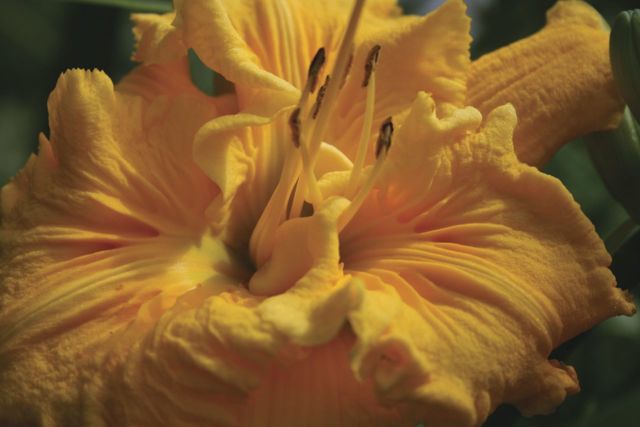
**A modern *Hemerocallis* cultivar, ‘Song of The Empire’, showing the effect of selection for ‘carving’ by alterations to petal tissue growth**.

**Fig. 10:**
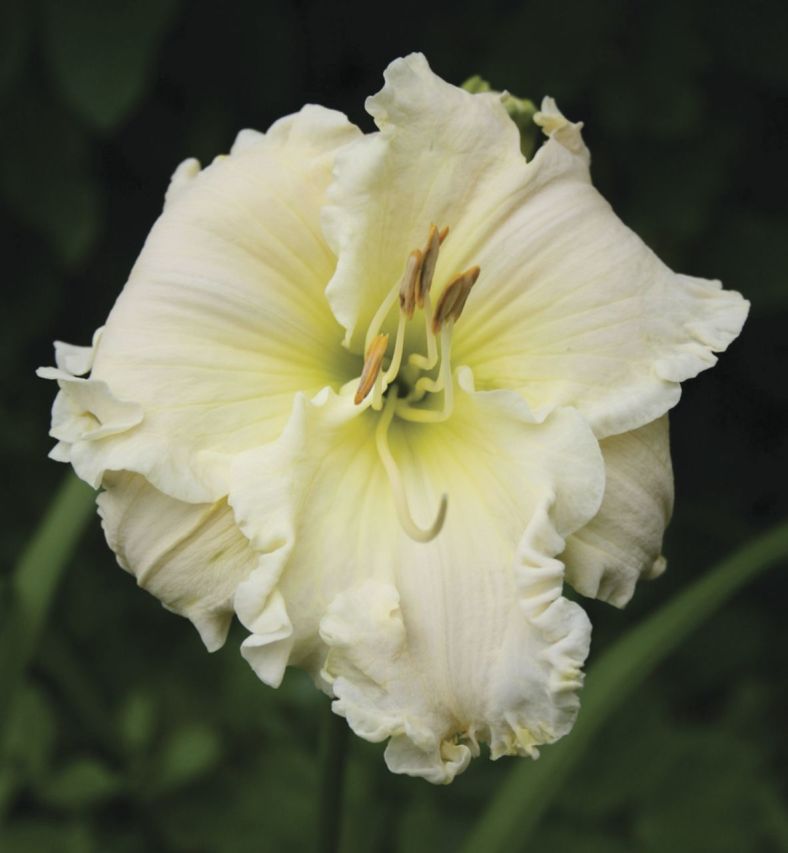
**A modern *Hemerocallis* cultivar, ‘Lacy All Over’, showing the effect of selection for ornate, ‘ruffled’ petal edges**.

**Fig. 11:**
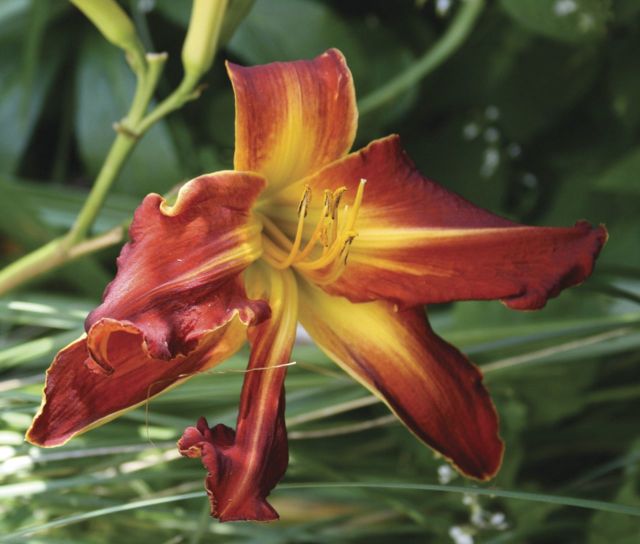
**A modern *Hemerocallis* hybrid (unnamed) showing the effect of selection for changes to the structure of the tepals to give the ‘sculptural’ effect of ‘pleating’**.

This decline in genetic diversity is concomitant with an *expansion* in phenotypic diversity—which may appear paradoxical unless the view is adopted that *de novo* heritable epigenetic variation can be selected and makes an important contribution to phenotypic variation in modern hybrids (Grant-Downton and Dickinson 2006). As might be expected, the changes are most prevalent in floral characteristics, the direct target of most selective processes. Today, over a century since the first artificial *Hemerocallis* hybrid was produced in England, the variation in floral characteristics is extraordinary. Darwin used the artificial selection of a multiplicity of fancy pigeons from ancestral rock pigeons to illustrate his ideas on strong selective forces being able to change the phenotypes of organisms ‘by descent’ (Darwin 1868). In *Hemerocallis*, plant scientists have an extraordinary untapped resource that has unique potential for exploitation by a new generation of plant geneticists and molecular biologists, as the original parental material is still retained and well documented in cultivation and herbaria. To change the aesthetics of the flower, breeders have selected variants not only in size, shape and dimensions, but also in flower colour, flower patterning and the number of floral organs (such as the so-called ‘polytepals’ with >3 organs per whorl). Other complex developmental changes have occurred to tepals in modern hybrids such as three-dimensional physical outgrowths on the tepal edges and surfaces (such as spine- and horn-like outgrowths) (Fig. 8) and alterations to normal floral tissue development to generate flowers with the appearance of surface ‘carving’ (Fig. 9), and other extreme physical ornamentations such as ruffling (Fig. 10) and ‘sculptural’ folding into novel shapes (Fig. 11). These extreme forms are largely restricted to the tetraploids at present. Such hybrids are, of course, interfertile with other hybrids of the same ploidy with simple ‘wild-type’ floral forms. Outside of specialist circles of collectors, these extraordinary products of intensive breeding are almost unknown, yet they could provide fascinating subjects for scientific study. Even the basic histology and developmental biology are yet to be investigated. Such studies would complement recent work on dicots, chiefly *Primula* (primrose) cultivars with aberrant floral development (Webster and Gilmartin 2003; Li *et al.* 2008, 2010).

**Fig. 12:**
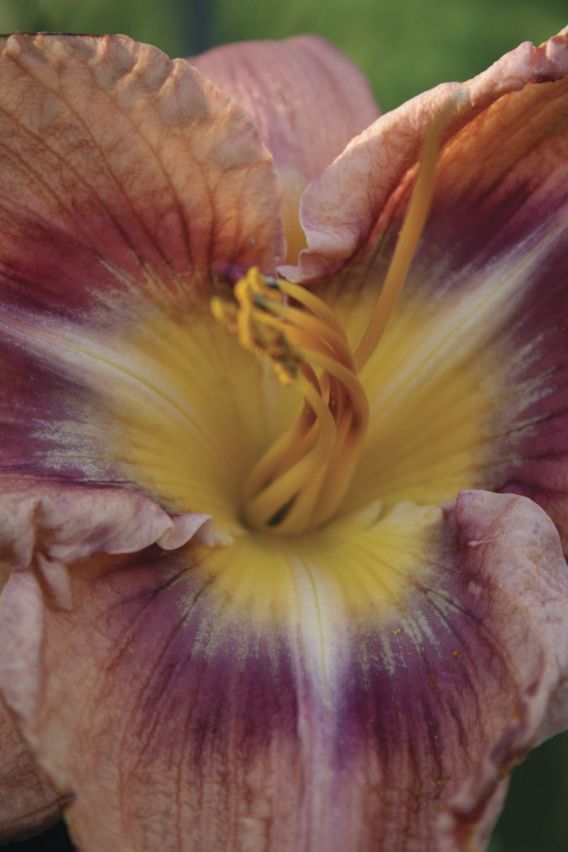
A modern *Hemerocallis* hybrid (unnamed) showing a region within the petal tissue where there is a marked ‘metallic’ or ‘reflective’ effect indicative of the development of structural colour.

It has already been proposed that horticultural collections of wind orchids (*Neofinetia falcata*) would make a useful resource for studies of floral development (Duttke *et al.* 2012). *Hemerocallis* provides another, possibly richer, source of developmental novelty to explore. For instance, enthusiasts have selected cultivars with complex patterned colour effects within the flower and, at least in some cases, it is likely that these effects are derived from structural colour as well as conventional pigmentation effects. Thus, modern varieties (such as ‘Visual Intrigue’ and ‘Time In A Bottle’) have narrow areas within the petal tissue where there is a marked ‘reflective’ or ‘metallic’ effect suggestive of the development of structural colour (Fig. 12). There would seem to be no similar or immediately obvious structural colour effects in the species and perhaps this trait has only recently been generated *de novo* by human selection. Given the great importance of structural colour in wild plants (Glover and Whitney 2010), these remarkable cultivars deserve to be investigated to determine the cellular and physical basis.

## Practicalities as a model plant

The great expansion of horticultural interest in this genus has left a valuable foundation legacy upon which its development as a ‘model’ organism could be initiated. *Hemerocallis* is easily cultivated and readily grown in the field and, more importantly, in controlled conditions in glasshouses and growth rooms. Plant breeders have selected particularly small-growing varieties such as ‘Stella D'Oro’ that would be ideal for use in scientific glasshouses and growth rooms where space is limited. Our own personal observations, from testing just a limited number of named cultivars, indicate that it is easy to identify genotypes (in our trial, the cultivars ‘Lavender Curls’ and ‘Teenie Girl’) that are able to grow uninterrupted and produce inflorescences throughout the year under the constant, controlled greenhouse conditions used for *A. thaliana* production*.* It is clear that even in a small sample of genotypes there is significant heterogeneity in response to cultivation under such conditions, and it would appear that seasonal changes to temperature (such as chilling and diurnal temperature fluctuations) and the intensity or duration of light may be important triggers for inflorescence initiation in certain genotypes. In itself, this area is worthy of further experimental work.

The horticultural interest in the genus has generated much valuable information on the practical aspects of cultivation, from sexual reproduction to clonal propagation. For instance, the mechanism of seed dormancy has been investigated (Griesbach and Voth 1957), and under controlled conditions any dormancy is easily and consistently broken by moist stratification in cold conditions (c. 5 °C) for 3–4 weeks followed by elevation to a higher temperature (c. 15–25 °C). Although seed viability is not retained for much longer than 12 months under conventional storage conditions (R. T. Grant-Downton, pers. obs.), long-term cryopreservation to maintain viability appears possible (Kholina and Voronkova 2008).

The desire for multiplication of selected cultivars of horticultural value has elicited the development of systems for clonal propagation by tissue culture. A significant load of endophytic microorganisms in *Hemerocallis* (Leifert and Cassells 2001) has, to date, effectively restricted this to callus-based systems. However, the genus can be susceptible to somaclonal variation by this method of propagation (Dunwell 1996; Podwyszyńska *et al.* 2010). Recent developments permit efficient callus initiation from small young leaf segments and subsequent shoot regeneration (Li, Z. *et al.* 2010). It has even been possible to make liquid suspension cultures of undifferentiated *Hemerocallis* cells and subsequently regenerate new plants from such material (Krikorian and Kann 1981), with the formation of embryogenic callus (Smith and Krikorian 1991). As might be expected, the amenability to tissue culture has assisted the development of systems for generating protoplasts and subsequent plant regeneration (Fitter and Krikorian 1981) and, most critically, transformation by particle bombardment (Aziz *et al.* 2003). Transformation is exceptionally important as it permits reverse genetics, for example using specific gene silencing ‘knockdown’ technology such as hairpin RNAi and artificial microRNA. It also permits other useful developments such as the identification of cell- and tissue-specific promoters by promoter traps, insertional mutagenesis using T-DNA and expression of fluorescent molecules such as green fluorescent protein.

One major barrier to adopting *Hemerocallis* as a model organism has been its significant genome size. The diploid hybrids have a genome size of 4522 Mbp/1C—comparable with barley (Tomkins *et al.* 2001). Today, the astonishing advances in high-throughput sequencing technology promise to overcome this inconvenience and propel studies of the genus into a new era where experimental investigations can be much better informed by and supported by genome sequence and gene expression data. Even basic genetics of the genus has recently moved forward with a study by Fogaça *et al.* (2012), where heritability of plant traits such as plant height and the number of floral buds per plant was assessed in modern hybrids. Adopting this kind of approach to studying other characteristics in hybrids would be most illuminating. However, the temporal limitations of using *Hemerocallis* in genetics studies remain daunting—each generation taking many months from seed formation through to flowering.

## Conclusions and forward look

At many different levels in plant science research, *Hemerocallis* offers superb potential as a model plant of the future. Whilst expensive, fundamental studies that rely on ‘omics’ approaches are likely to be able to explore only one or two different members of the genus, other approaches using more traditional molecular biology, genetics, biochemistry and microscopy techniques will not be subject to such restrictions and can sample the full spectrum of diversity, from diploid species to the most intensely selected man-made hybrid tetraploids. New insights into fascinating subjects such as PCD, SI systems and the cellular, molecular and genetic basis of morphological innovations could be generated by exploration in this genus. Equally, more applied studies, such as identifying and studying molecules of potential biomedical importance, would be assisted by embracing *Hemerocallis* as a model organism. We look forward to a future where many more plant biologists are not only aware of, but also actively utilizing, *Hemerocallis* in research.

## Sources of funding

M.J.R.-E. has been financially supported by the ‘Movilidad del Profesorado’ (PR2007-0487) Initiative, MEC, Spain and ‘Beca de Movilidad del Gobierno de Canarias’. R.T.G-D. would also like to thank support from the scheme ‘Estancias Profesores Invitados, Programa de Apoyo a la Investigacion de la Universidad de la Laguna Vicerrectorado de Investigacion y Transferencia de Conocimiento.’ The authors would like to thank the British Hosta and Hemerocallis Society and the Stanley Smith (UK) Horticultural Trust for small grant funding to pursue research on the genus *Hemerocallis.*

## Contributions by the authors

Both authors contributed to researching and writing the manuscript.

## Conflicts of interest statement

None declared.

## References

[PLS055C1] Angiosperm Phylogeny Group (APG) (1998). An ordinal classification for the families of flowering plants. Annals of the Missouri Botanical Garden.

[PLS055C2] Angiosperm Phylogeny Group (APG) (2003). An update of the angiosperm phylogeny group classification for the orders and families of flowering plants: APG II. Botanical Journal of the Linnean Society.

[PLS055C3] Angiosperm Phylogeny Group (APG) (2009). An update of the angiosperm phylogeny group classification for the orders and families of flowering plants: APG III. Botanical Journal of the Linnean Society.

[PLS055C4] Arisumi T (1964). Colchicine-induced tetraploid and cytochimeral daylilies. Journal of Heredity.

[PLS055C5] Arisumi T (1973). Embryo development and seed set in crosses for triploid day lilies. Botanical Gazette.

[PLS055C6] Aziz AN, Sauve RJ, Zhou S (2003). Genetic transformation of Stella De Oro daylily by particle bombardment. Canadian Journal of Plant Science.

[PLS055C7] Bieleski RL (1995). Onset of phloem export from senescent petals of daylily. Plant Physiology.

[PLS055C8] Bieleski RL, Reid MS (1992). Physiological changes accompanying senescence in the ephemeral daylily flower. Plant Physiology.

[PLS055C9] Bor JY, Chen HY, Yen GC (2006). Evaluation of antioxidant activity and inhibitory effect on nitric oxide production of some common vegetables. Journal of Agricultural and Food Chemistry.

[PLS055C10] Brenchley R, Spannagl M, Pfeifer M, Barker GLA, D/'Amore R, Allen AM, McKenzie N, Kramer M, Kerhornou A, Bolser D, Kay S, Waite D, Trick M, Bancroft I, Gu Y, Huo N, Luo M-C, Sehgal S, Gill B, Kianian S, Anderson O, Kersey P, Dvorak J, McCombie WR, Hall A, Mayer KFX, Edwards KJ, Bevan MW, Hall N (2012). Analysis of the bread wheat genome using whole-genome shotgun sequencing. Nature.

[PLS055C11] Chandler C (1940). Microsporogenesis in triploid and diploid plants of *Hemerocallis fulva*. Bulletin of the Torrey Botanical Club.

[PLS055C12] Chung MG, Kang SS (1994). *Hemerocallis hongdoensis* (Liliaceae): a new species from Korea. Novon.

[PLS055C13] Cichewicz RH, Nair MG (2002). Isolation and characterization of stelladerol, a new antioxidant naphthalene glycoside, and other antioxidant glycosides from edible daylily (*Hemerocallis*) flowers. Journal of Agricultural and Food Chemistry.

[PLS055C14] Cichewicz RH, Lim K-C, McKerrow JH, Nair MG (2002). Kwanzoquinones A–G and other constituents of *Hemerocallis fulva* ‘Kwanzo’ roots and their activity against the human pathogenic trematode *Schistosoma mansoni*. Tetrahedron.

[PLS055C15] Cichewicz RH, Zhang Y, Seeram NP, Nair MG (2004). Inhibition of human tumor cell proliferation by novel anthraquinones from daylilies. Life Sciences.

[PLS055C16] Dahlgren RMT, Clifford HT, Yeo PF (1985). The families of the monocotyledons.

[PLS055C17] Darwin C (1868). Variation in animals and plants under domestication..

[PLS055C18] Deschamps S, Campbell M (2010). Utilization of next-generation sequencing platforms in plant genomics and genetic variant discovery. Molecular Breeding: New Strategies in Plant Improvement.

[PLS055C19] Dunwell WC (1996). *Hemerocallis* (daylily) propagation. Proceedings of the International Plant Propagators’ Society.

[PLS055C20] Duttke S, Zoulias N, Kim M (2012). Mutant flower morphologies in the wind orchid, a novel orchid model species. Plant Physiology.

[PLS055C21] Ehrenfeld J (2008). Exotic invasive species in urban wetlands: environmental correlates and implications for wetland management. Journal of Applied Ecology.

[PLS055C22] Fitter MS, Krikorian AD (1981). Recovery of totipotent cells and plantlet production from daylily protoplasts. Annals of Botany.

[PLS055C23] Fogaça LA, Oliveira RA, Cuquel FL, Filho JCB, Vendrame WA, Tombolato AFC (2012). Heritability and genetic correlation in daylily selection. Euphytica.

[PLS055C24] Franklin-Tong NV, Franklin FC (2003). Gametophytic self-incompatibility inhibits pollen tube growth using different mechanisms. Trends in Plant Science.

[PLS055C25] Glover BJ, Whitney HM (2010). Structural colour and iridescence in plants: the poorly studied relations of pigment colour. Annals of Botany.

[PLS055C26] Grant-Downton RT, Dickinson HG (2006). Epigenetics and its implications for plant biology 2. The ‘epigenetic epiphany’: epigenetics, evolution and beyond. Annals of Botany.

[PLS055C27] Greenberg GT (1997). Programmed cell death in plant-pathogen interactions. Annual Review of Plant Physiology and Plant Molecular Biology.

[PLS055C28] Griesbach RA, Voth PD (1957). On dormancy and seed germination in *Hemerocallis*. Botanical Gazette.

[PLS055C29] Gu L, Liu Y-J, Wang Y-B, Yi L-T (2012). Role for monoaminergic systems in the antidepressant-like effect of ethanol extracts from *Hemerocallis citrina*. Journal of Ethnopharmacology.

[PLS055C30] Guerrero C, de la Calle M, Reid MS, Valpuesta V (1998). Analysis of the expression of two thiolprotease genes from daylily (*Hemerocallis* spp.) during flower senescence. Plant Molecular Biology.

[PLS055C31] Hasegawa M, Yahara T, Yasumoto A, Hotta M (2006). Bimodal distribution of flowering time in a natural hybrid population of daylily (*Hemerocallis fulva*) and nightlily (*Hemerocallis citrina*). Journal of Plant Research.

[PLS055C32] Hirota SK, Nitta K, Kim Y, Kato A, Kawakubo N, Yasumoto AA, Yahara T (2012). Relative role of flower color and scent on pollinator attraction: experimental tests using F_1_ and F_2_ hybrids of daylily and nightlily. PLoS One.

[PLS055C33] Kang SS, Chung MG (1997). *Hemerocallis taeanensis* (Liliaceae), a new species from Korea. Systematic Botany.

[PLS055C34] Kawano S (1961). On a natural hybrid population of *Hemerocallis*. Canadian Journal of Botany.

[PLS055C35] Kholina AB, Voronkova NM (2008). Conserving the gene pool of Far Eastern plants by means of seed cryopreservation. Biology Bulletin.

[PLS055C36] Konar RN, Stanley RG (1969). Wall-softening enzymes in the gynoecium and pollen of *Hemerocallis fulva*. Planta.

[PLS055C37] Krikorian AD, Kann RP (1981). Plantlet production from morphogenetically competent cell suspensions of daylily. Annals of Botany.

[PLS055C38] Leifert C, Cassells AC (2001). Microbial hazards in plant tissue and cell cultures. In Vitro Cellular & Developmental Biology – Plant.

[PLS055C39] Li J, Webster M, Dudas B, Cook H, Manfield I, Davies B, Gilmartin PM (2008). The *S* locus-linked *Primula* homeotic mutant *sepaloid* shows characteristics of a B-function mutant but does not result from mutation in a B-function gene. The Plant Journal.

[PLS055C40] Li J, Dudas B, Webster MA, Cook HE, Davies BH, Gilmartin PM (2010). *Hose in Hose*, an *S* locus-linked mutant of *Primula vulgaris*, is caused by an unstable mutation at the *Globosa* locus. Proceedings of the National Academy of Sciences of the USA.

[PLS055C41] Li Z, Mize K, Campbell F (2010). Regeneration of daylily (*Hemerocallis*) from young leaf segments. Plant Cell, Tissue and Organ Culture.

[PLS055C42] Li ZW, Pinkham L, Campbell NF, Espinosa AC, Conev R (2009). Development of triploid daylily (*Hemerocallis*) germplasm by embryo rescue. Euphytica.

[PLS055C43] Lin YL, Lu CK, Huang YJ, Chen HJ (2011). Antioxidative caffeoylquinic acids and flavonoids from *Hemerocallis fulva* flowers. Journal of Agricultural and Food Chemistry.

[PLS055C44] Mahagamasekera MGP, Leung DWM (2001). Development of leucine aminopeptidase activity during daylily flower growth and senescence. Acta Physiologiae Plantarum.

[PLS055C45] Mlček J, Rop O (2011). Fresh edible flowers of ornamental plants—a new source of nutraceutical foods. Trends in Food Science and Technology.

[PLS055C46] Mulcahy DL (1979). The rise of the angiosperms: a genecological factor. Science.

[PLS055C47] Nitta K, Yasumoto AA, Yahara T (2010). Variation of flower opening and closing times in F_1_ and F_2_ hybrids of daylily (*Hemerocallis fulva*; Hemerocallidaceae) and nightlily (*H. citrina*). American Journal of Botany.

[PLS055C48] Noguchi J, Hong DY (2004). Multiple origins of the Japanese nocturnal *Hemerocallis citrina* var. *vespertina* (Asparagales: Hemerocallidaceae): evidence from noncoding chloroplast DNA sequences and morphology. International Journal of Plant Sciences.

[PLS055C49] Ossowski S, Schwab R, Weigel D (2008). Gene silencing in plants using artificial microRNAs and other small RNAs. The Plant Journal.

[PLS055C50] Panavas T, Rubinstein B (1998). Oxidative events during programmed cell death of daylily (*Hemerocallis* hybrid) petals. Plant Science.

[PLS055C51] Panavas T, Reid PD, Rubinstein B (1998). Programmed cell death of daylily petals: activities of wall-based enzymes and effects of heat shock. Plant Physiology and Biochemistry.

[PLS055C52] Panavas T, LeVangie R, Mistler J, Reid PD, Rubinstein B (2000). Activities of nucleases in senescing daylily petals. Plant Physiology and Biochemistry.

[PLS055C53] Parish RW, Li SF (2010). Death of a tapetum: a programme of developmental altruism. Plant Science.

[PLS055C54] Petit TL, Peat JP (2000). The color encyclopedia of daylilies.

[PLS055C55] Petit TL, Peat JP (2004). The daylily: a guide for gardeners.

[PLS055C56] Podwyszyńska M, Gabryszewska E, Korbin M, Jasiński A (2010). Somaclonal variation in micropropagated *Hemerocallis* sp. plants determined by phenotype and molecular markers, RAPA die d and ISSR. Biotechnologia.

[PLS055C57] Pyek P (2003). How reliable are data on alien species in Flora Europaea?. Flora.

[PLS055C58] Rop O, Mlcek J, Jurikova T, Neugebauerova J, Vabkova J (2012). Edible flowers—a new promising source of mineral elements in human nutrition. Molecules.

[PLS055C59] Seberg O, Petersen G, Davis JI, Pires JC, Stevenson DW, Chase MW, Fay MF, Devey DS, Jorgensen T, Sytsma KJ, Pillon Y (2012). Phylogeny of the Asparagales based on three plastid and two mitochondrial genes. American Journal of Botany.

[PLS055C60] Smith DL, Krikorian AD (1991). Growth and maintenance of an embryogenic cell culture of daylily (*Hemerocallis*) on hormone-free medium. Annals of Botany.

[PLS055C61] Stephenson P, Rubinstein B (1998). Characterization of proteolytic activity during senescence in daylilies. Physiologia Plantarum.

[PLS055C62] Stout AB (1934). Daylilies: the wild species and garden clones, both old and new, of the genus Hemerocallis.

[PLS055C63] Stout AB, Chandler C (1933). Pollen-tube behavior in *Hemerocallis* with special reference to incompatibilities. Bulletin of the Torrey Botanical Club.

[PLS055C64] Taylor LP, Hepler PK (1997). Pollen germination and tube growth. Annual Review of Plant Physiology and Plant Molecular Biology.

[PLS055C65] Tomkins JP, Wood TC, Barnes LS, Westman A, Wing RA (2001). Evaluation of genetic variation in the daylily (*Hemerocallis* spp.) using AFLP markers. Theoretical and Applied Genetics.

[PLS055C66] Traub HP (1949). Colchicine poisoning in relation to *Hemerocallis* and some other plants. Science.

[PLS055C67] Uezu E (1998). Effects of *Hemerocallis* on sleep in mice. Psychiatry and Clinical Neurosciences.

[PLS055C68] Valder P (1999). The garden plants of China.

[PLS055C69] Valpuesta V, Lange NE, Guerrero C, Reid MS (1995). Up-regulation of a cysteine protease accompanies the ethylene-insensitive senescence of daylily (*Hemerocallis*) flowers. Plant Molecular Biology.

[PLS055C70] Wang J-H, Humphreys DJ, Stodulski GBJ, Middleton DJ, Barlow RM, Lee JB (1989). Structure and distribution of a neurotoxic principle, hemerocallin. Phytochemistry.

[PLS055C71] Webb CO, Ackerly DD, Kembel SW (2008). Phylocom: software for the analysis of phylogenetic community structure and trait evolution. Bioinformatics.

[PLS055C72] Webb DA, Tutin TG, Heywood VH, Burges NA, Moore DM, Valentine DH, Walters SM, Webb DA (1980). Hemerocallis. Flora Europaea Volume 5 Alismataceae to Orchidaceae.

[PLS055C73] Webster MA, Gilmartin PA (2003). A comparison of early floral ontogeny in wild-type and floral homeotic mutant phenotypes of *Primula*. Planta.

[PLS055C74] Weinthal D, Tovkach A, Zeevi V, Tzfira T (2010). Genome editing in plant cells by zinc finger nucleases. Trends in Plant Science.

[PLS055C75] Xiao SH, Keiser J, Chen MG, Tanner M, Utzinger J (2010). Research and development of antischistosomal drugs in the People's Republic of China a 60-year review. Advances in Parasitology.

[PLS055C76] Xiaobai J (1996). The chromosomes of *Hemerocallis* (Liliaceae). Kew Bulletin.

[PLS055C77] Yasumoto AA, Yahara T (2006). Post-pollination reproductive isolation between diurnally and nocturnally flowering daylilies, *Hemerocallis fulva* and *Hemerocallis citrina* (Hemerocallidaceae). Journal of Plant Research.

[PLS055C78] Yasumoto AA, Yahara T (2008). Reproductive isolation on interspecific backcross of F_1_ pollen to parental species, *Hemerocallis fulva* and *H. citrina* (Hemerocallidaceae). Journal of Plant Research.

[PLS055C79] Yi LT, Li J, Li HC, Zhou Y, Su BF, Yang KF, Jiang M, Zhang YT (2012). Ethanol extracts from *Hemerocallis citrina* attenuate the decreases of brain-derived neurotrophic factor, TrkB levels in rat induced by corticosterone administration. Journal of Ethnopharmacology.

[PLS055C80] Zhang Y, Cichewicz RH, Nair MG (2004). Lipid peroxidation inhibitory compounds from daylily (*Hemerocallis fulva*) leaves. Life Science.

